# MamA essentiality in *Mycobacterium smegmatis* is explained by the presence of an apparent cognate restriction endonuclease

**DOI:** 10.1186/s13104-020-05302-z

**Published:** 2020-09-29

**Authors:** Samantha E. Randall, Maria Carla Martini, Ying Zhou, Samantha R. Joubran, Scarlet S. Shell

**Affiliations:** 1 Department of Biology & Biotechnology, Worcester Polytechnic Institute, Worcester, MA USA; 2 Program in Bioinformatics & Computational Biology, Worcester Polytechnic Institute, Worcester, MA USA

**Keywords:** *Mycobacterium smegmatis*, MamA, Restriction-Modification system, DNA methylation, Methyltransferase

## Abstract

**Objective:**

Restriction-Modification (R-M) systems are ubiquitous in bacteria and were considered for years as rudimentary immune systems that protect bacterial cells from foreign DNA. Currently, these R-M systems are recognized as important players in global gene expression and other cellular processes such us virulence and evolution of genomes. Here, we report the role of the unique DNA methyltransferase in *Mycobacterium smegmatis*, which shows a moderate degree of sequence similarity to MamA, a previously characterized methyltransferase that affects gene expression in *Mycobacterium tuberculosis* and is important for survival under hypoxic conditions.

**Results:**

We found that depletion of *mamA* levels impairs growth and produces elongated cell bodies. Microscopy revealed irregular septation and unevenly distributed DNA, with large areas devoid of DNA and small DNA-free cells. Deletion of MSMEG_3214, a predicted endonuclease-encoding gene co-transcribed with *mamA*, restored the WT growth phenotype in a *mamA*-depleted background. Our results suggest that the *mamA*-depletion phenotype can be explained by DNA cleavage by the apparent cognate restriction endonuclease MSMEG_3214. In addition, in silico analysis predicts that both MamA methyltransferase and MSMEG_3214 endonuclease recognize the same palindromic DNA sequence. We propose that MamA and MSMEG_3214 constitute a previously undescribed R-M system in *M. smegmatis*.

## Introduction

The primary role of bacterial Restriction-Modification (R-M) systems has typically been viewed as protection from foreign DNA, such as plasmids and phages. However, further work revealed these systems can have other important functions, including regulation of gene expression and promoting bacterial evolution by enhancing DNA recombination [1–4].

To date, no complete R-M systems have been defined in *M. tuberculosis*, the causative agent of tuberculosis, or in its non-pathogenic relative *M. smegmatis*, a model system widely used to study the basic biology of *M. tuberculosis*. We previously identified and characterized one of the three predicted DNA methyltransferases in *M. tuberculosis*, MamA [5], which is non-essential for *M. tuberculosis* survival in vitro and during infection [6–8]. We and others showed that MamA is an adenine methyltransferase that affects expression of a number of genes in *M. tuberculosis* during log phase growth and promotes survival under hypoxic conditions [5, 9]. However, the function of its homolog in *M. smegmatis* has not been shown. Unlike in *M. tuberculosis*, the *mamA* homolog in *M. smegmatis* (MSMEG_3213) is essential for in vitro growth. Several attempts in our laboratory failed to obtain *mamA* knockouts, which is consistent with recently reported Tn-seq data [10]. Here, we demonstrate that *mamA* and its co-transcribed restriction endonuclease form a previously undescribed R-M system in *M. smegmatis*.

## Main text

### Materials and methods

#### Strains and growth conditions

*M. smegmatis* mc^2^155 strains were grown in Middlebrook 7H9 media (Sigma) supplemented with ADC [11]. When needed, Middlebrook 7H10 solid media (Sigma) supplemented with ADC was used. Cells were cultured at 37 **°**C at 200 rpm. When required, 50 μg/mL kanamycin or 250 μg/mL hygromycin were added.

Inducible *mamA* knockdown (KD) was achieved by modification of the L5-integrating CRISPRi plasmid pJR962 [11], which contains a dCas9 and a non-targeting sgRNA. The non-targeting sgRNA was replaced with 20 nts of the *mamA* (MSMEG_3213) coding sequence (MSMEG_3213 coordinates: 103-122). The rescue plasmid containing full *mamA* gene sequence with two point mutations at nucleotides 96 (C → G) and 99 (C → T) of the coding sequence was done in the pSS047 backbone, which integrates at the Giles *attB* site and is derived from pGH1000A [12] using the native *mamA* promoter and 5’ UTR (242 nt upstream of the coding sequence: 42 nt known UTR and 200 nt predicted promoter). The MSMEG_3214 knockout was obtained by recombineering [13] in which nucleotides 29-993 of the annotated MSMEG_3214 coding sequence were replaced by a hygromycin resistance cassette.

#### Growth curve determination

*mamA* KD and overexpressing strains were grown to log phase and diluted in 7H9 media to an OD_600nm_ of 0.01 or 0.001 and 200 μL aliquots were placed in 96 well plates. Growth was determined by automated OD recording in an Epoch 2 plate reader (BioTek). Samples were incubated at 37 °C with shaking using a single orbital pattern at 807 cpm. OD_600nm_ was measured every 10 min for 24 h. When indicated, Anhydrotetracycline (ATc) was added to a final concentration of 200 ng/mL for induction of dCas9 and sgRNA expression.

#### Viability assay

Log-phase cultures were normalized and diluted to an OD_600nm_ of 0.01 as described above. When indicated, ATc was added. For CFU determination, samples were taken at the indicated times and serial dilutions were plated on antibiotic-free 7H10 plates.

#### Brightfield and fluorescence microscopy

Cultures were grown in the presence or absence of ATc to an OD_600nm_ of 0.05-0.15. Aliquots of 1.5 mL were pelleted and the supernatant removed. Pellets were resuspended in 500 μL of 2% paraformaldehyde in PBS and incubated at room temperature for 30 min. Cells were then pelleted and resuspended in 900 μL of PBS + 0.1% Tween 20 (PBS-T). This was repeated for a total of two washes. Pellets were finally resuspended in 50 μL of PBS-T. Fixed cells were then stained with SYTO 24 and/or FM 4-64FX (both ThermoFisher). For DNA visualization, cells were stained in 2 μM SYTO 24 for 10 min before washing and resuspending in 50 μL of PBS-T. For membrane staining, cells were stained with 8 μL of 100 μg/mL FM 4-64FX for 10 min before washing twice with PBS-T and resuspending in 100 μL of PBS-T. 2 μL of cells were mixed with 6 μL of mounting media (20 mM Tris pH 8, 0.5% N-propyl gallate, and 50% glycerol), and pipetted onto an agar pad on a slide. Cells were viewed using a Zeiss AX10 microscope with ApoTome and a 40X oil objective.

Analysis of microscopy photos was performed using ImageJ (NIH) and GraphPad Prism 8 software. Statistical analysis was performed using one-way ANOVA, Dunn’s multiple comparison tests, and non-parametric tests.

### Results

#### Depletion of MamA impairs growth and reduces viability in *M. smegmatis*

**Fig. 1:**
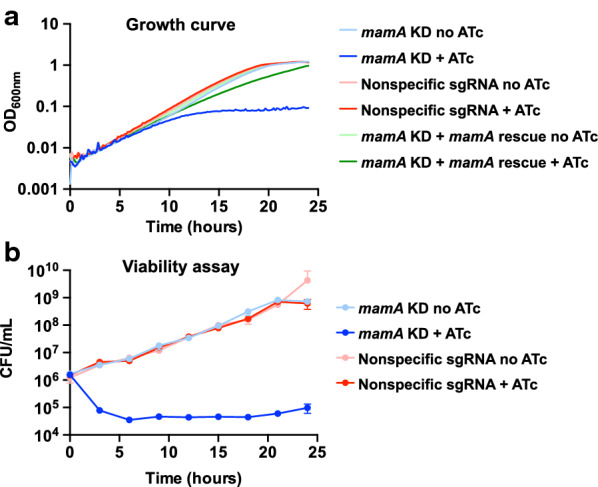
*mamA* depletion causes growth cessation in *M. smegmatis*. **a** Strains with depleted *mamA* (*mamA* KD), nonspecific CRISPRi control with a sgRNA sequence that does not target any specific part of *M. smegmatis* genome (Nonspecific sgRNA), or a rescue vector containing a synonymous *mamA* mutant that is not bound by dCas9 (*mamA* Rescue) were incubated in 7H9 in presence (+) or absence of ATc to activate or not the CRISPRi knockdown system, respectively. Three biological replicates of *mamA* KD, four biological replicates of *mamA* Rescue, and two technical replicates of each were used and values were averaged. **b** The strains were grown in 7H9 and CFUs were calculated at different timepoints

We generated an ATc-inducible *mamA* KD strain and a control with a non-specific CRISPRi construct containing an sgRNA that does not target the *M. smegmatis* genome [11]. We first evaluated growth of *mamA* KD in 7H9 medium. Consistent with the idea that *mamA* is essential for growth, we observed a slowing of growth followed by growth cessation in the *mamA* KD strain after 10 h incubation in presence of ATc (Fig. 1a).

We previously showed that *mamA* is co-transcribed with its downstream gene, MSMEG_3214 [14]. As CRISPRi represses transcription of co-transcribed genes, we constructed a rescue vector expressing a second copy of *mamA* with two synonymous point mutations that disrupt the PAM adjacent to the sgRNA binding site and thus prevent dCas9 binding. This vector was integrated into the genome of *M. smegmatis* that also contained the *mamA* KD construct. We observed that, in presence of ATc, the rescued strain recovered the ability to grow normally (Fig. 1a), confirming that the growth cessation observed in the *mamA* KD strain is not attributable to depletion of the downstream gene.

To determine whether the cells were viable after *mamA* KD, we calculated CFUs at different timepoints (Fig. 1b). Strikingly, the *mamA* depleted cultures displayed a nearly 100-fold decrease in CFUs within the first 6 h of ATc addition, and the number of viable cells remained similar during the experiment, suggesting that the depletion of *mamA* results in cell death.

#### MamA depletion results in cell elongation and asymmetric DNA distribution

**Fig. 2:**
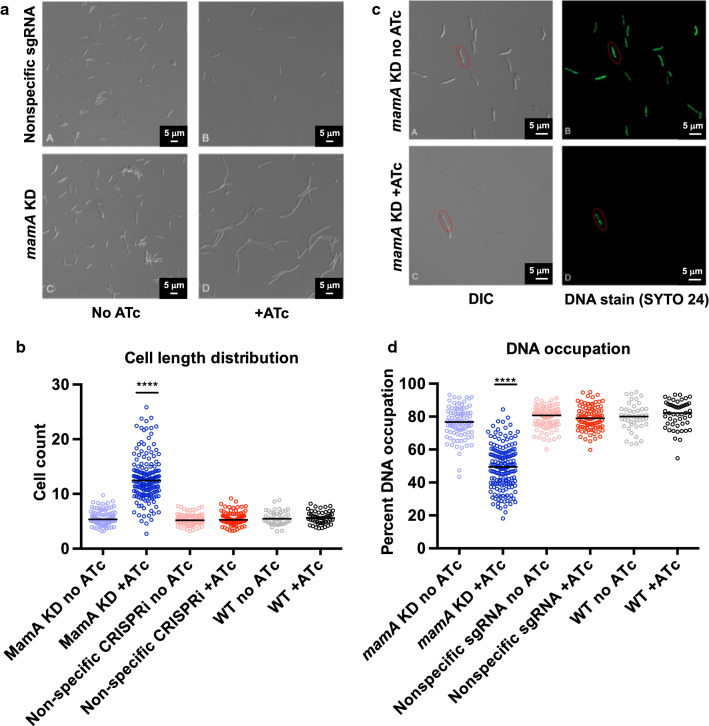
Lowering *mamA* levels leads to cell filamentation and asymmetric DNA distribution. **a** DIC Microscopy showing WT (upper panels and *mamA* KD no ATc) and *mamA* depleted (*mamA* KD +ATc) phenotypes. **b** Cell length distribution determined by microscopy and analyzed with ImageJ software. **c** DIC microscopy and DNA staining with SYTO 24 in *mamA* depleted (+ ATc) and non-depleted (no ATc) cells. **d** Quantification of DNA occupation observed in SYTO 24-stained cells. Percent DNA occupation was calculated by dividing length of DNA fluorescence by cell length. ****p < 0.0001 Kruskal–Wallis followed by Dunn’s multiple comparisons test

Considering the growth defect in *mamA* depleted cultures, we sought to investigate the impact of *mamA* KD on cell morphology. Cultures were grown in presence or absence of ATc for 12 h and viewed by DIC microscopy (Fig. 2a). *mamA* depleted cells (panel D) were elongated compared to those of the control cells. We measured cell lengths in a blinded analysis for each strain and condition (Fig. 2b). While control strains and treatments had an average length approximately 5.7 μm, *mamA* KD cells exhibited an average length of 12.8 μm. *mamA* KD cell lengths also had a larger coefficient of variation, 31.6%, compared to control cells, 21.6%. Reduction of *mamA* levels therefore causes filamentation in *M. smegmatis*.

We further determined DNA content and localization by fluorescence microscopy using the DNA stain SYTO 24 (Fig. 2c). Control cells showed fairly even distribution of DNA throughout the cell length, while *mamA* depleted cells displayed highly uneven distributions of DNA, with fluorescence often focused around the midpoint of the cell leaving large areas devoid of DNA. To quantify the difference in DNA distribution, we calculated DNA occupancy for cells as in [15]. Cells lengths and lengths of fluorescent zones were measured in ImageJ in a blinded analysis. *mamA* depleted cells had significantly lower DNA occupancy than control cells, with averages of 49.1% occupancy, and 79.3% occupancy, respectively (Fig. 2d).

#### MamA depleted cells have three major phenotypes

**Fig. 3:**
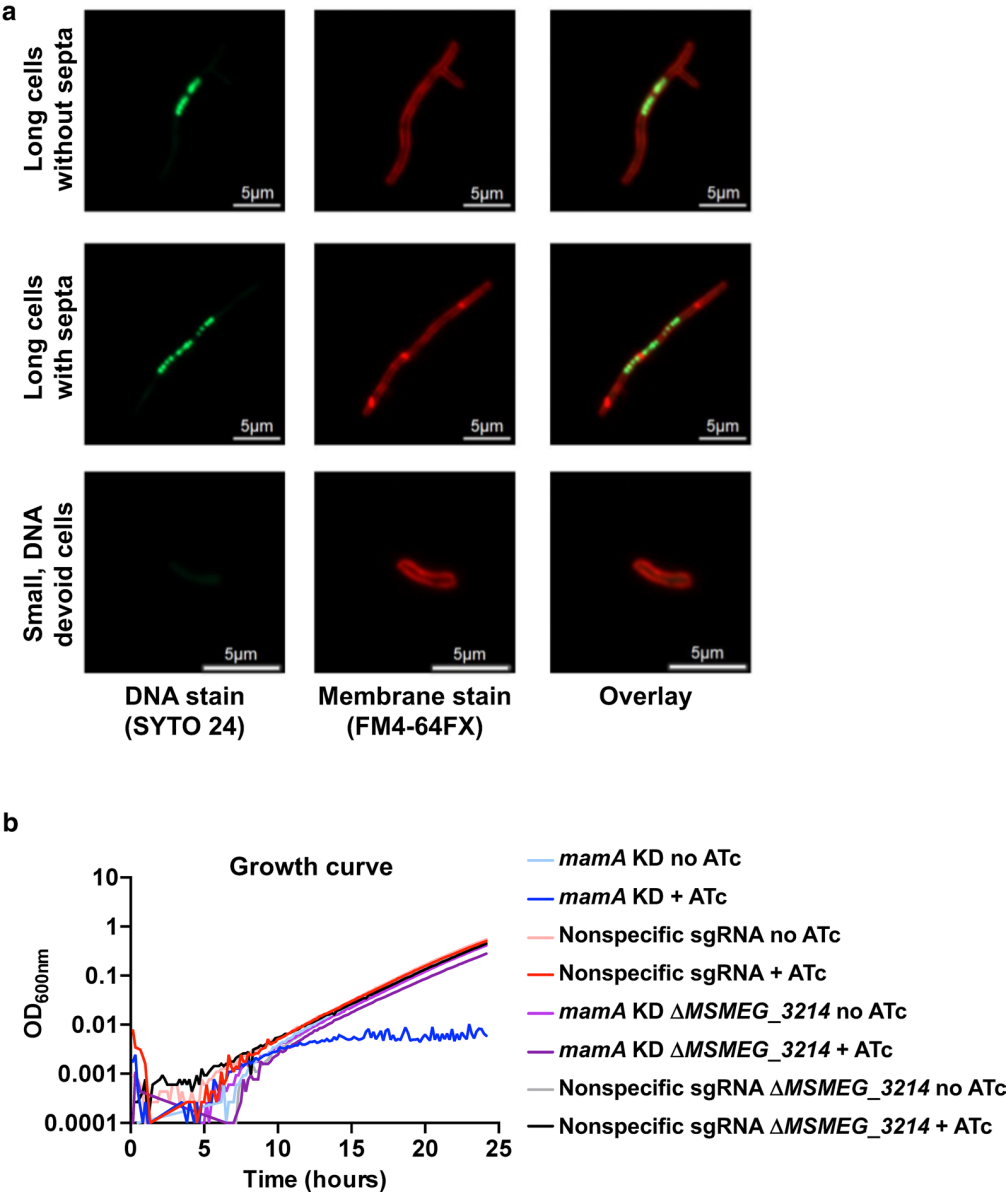
Morphologies of *mamA* depleted cells and rescue by deletion of MSMEG_3214. **a** Microscopy of the *mamA* KD strain treated with ATc for 12 h. Cells were stained with SYTO 24 (green) and FM 4-64FX (red) to visualize DNA and membranes, respectively. The images represent cells of three major phenotypic groups observed. The left column shows DNA stained only, the center column shows membrane stained only, and the right column shows the merged fluorescent images. **b** Strains with depleted *mamA* (*mamA* KD), nonspecific CRISPRi control with an sgRNA sequence that does not target any specific part of *M. smegmatis* genome (Nonspecific sgRNA), *mamA* KD in an MSMEG_3214-deleted background (*mamA* KD ΔMSMEG_3214), and the ΔMSMEG_3214 strain with a nonspecific sgRNA (Nonspecific sgRNA ΔMSMEG_3214) were incubated in 7H9 in presence (+) or in absence of ATc to activate or not the CRISPRi knockdown system, respectively

To determine if elongated *mamA* KD cells had septa (chaining phenotype) or not (filamenting phenotype), *mamA* KD cells were grown in presence of ATc for 12 h and double stained with the membrane stain FM 4-64FX and the DNA stain SYTO 24. We noted three distinct categories of *mamA* KD cells (Fig. 3a). The first were long cells with evidence of septation. The number of septa per cell varied from one to four within this category. Most of the chaining cells had DNA located within each segment; however, many had segments at the poles that did not appear to contain DNA. The second category, long cells without an obvious septum, were grouped by the appearance of cell filamentation. These cells tended to have DNA localized near the middle of the cell with large portions of the cells near the poles that were apparently devoid of DNA. The third category were small cells, which were similar in size or smaller than control cells, that had no evidence of DNA. These data show that *mamA* depletion leads to irregular cell division and DNA distribution.

#### MamA appears to be part of an R-M system in *M. smegmatis*

Considering the essentiality of *mamA* in *M. smegmatis*, we suspected that this methylase could be part of an R-M system, and that lack of MamA is toxic because an active restriction endonuclease recognizing the same sequence cleaves the genome when unmethylated. In addition, it has been observed that disturbance of R-M systems increases cell filamentation in bacteria [16–18], supporting the idea that the unbalance in the R-M system could be the reason for the *mamA* KD phenotype. We previously found that *mamA* is co-transcribed with the downstream gene, MSMEG_3214 [14]. A BLASTp of MamA and MSMEG_3214 sequences against REBASE, a comprehensive database of information about restriction enzymes and DNA methyltransferases involved in restriction-modification [19], revealed that these genes encode for a predicted Type II N4-cytosine or N6-adenine DNA methyltransferase, and a predicted Type II restriction enzyme, annotated as M.Msms1ORF2534P and Msms1ORF2534P, respectively, and both are predicted to recognize the same palindromic sequence, CTCGAG. Thus, we reasoned that, if MSMEG_3214 is the cognate restriction endonuclease of MamA and the phenotype observed in depleted MamA cells is due to self-DNA cleavage, a knockout of MSMEG_3214 in the MamA-depleted background should restore the WT phenotype. We generated an MSMEG_3214 deletion strain containing the *mamA* CRISPRi construct and evaluated its growth in the presence and absence of ATc (Fig. 3b). In the absence of MSMEG_3214, *mamA* KD does not affect growth, supporting the hypothesis that MamA and MSMEG_3214 are part of an active R-M system in *M. smegmatis*.

### Discussion

*M. smegmatis* is a widely used non-pathogenic model for *M. tuberculosis.* These species share numerous orthologous genes, but in most cases there is no experimental determination of whether orthologous genes pairs have equivalent functions in both organisms. While *M. tuberculosis* MamA is a DNA methyltransferase that affects gene expression during normal growth and supports survival in hypoxia [5], here we provide evidence that the orthologous methyltransferase in *M. smegmatis* is part of an R-M system. Although the catalytic activity of MamA in both species is DNA methylation, they have different biological functions, which may reflect different evolutionary pathways. Notably, *M. tuberculosis* is an obligate lung pathogen, and as such does not likely come into frequent contact with phages. Selective pressure for maintenance of R-M systems may therefore be reduced. Consistent with this, a type I R-M system (HsdM/HsdS) in *M. tuberculosis* is missing the cognate restriction enzyme. The different sequence specificities of MamA in *M. tuberculosis* and *M. smegmatis* [5, 19] also highlight their evolutionary and functional divergence. Interestingly, multiple *M. tuberculosis* sub-lineages have MamA mutations that cause complete or partial loss of function [5, 9, 20, 21].

A BLAST search against the NCBI database performed in our laboratory revealed that while MamA orthologs are present in most mycobacteria, MSMEG_3214 orthologs are absent from the most prominent pathogenic species including *M. tuberculosis*, *M. avium* complex, *M. leprae*, *M. abscessus*, *M. marinum*, and *M. kansasii*. Some fast-growing mycobacteria capable of causing disease do encode MSMEG_3214 orthologs; for example, *M. fortuitum*, *M. peregrinum*, and *M. goodii*.

This work provides evidence for the first time that an active R-M system is present in *M. smegmatis*. Further experiments need to be done to confirm the recognition of CTCGAG palindromic sequence by both *M. smegmatis* MamA methyltransferase and MSMEG_3214 endonuclease as predicted by REBASE database.

## Limitations

- The levels of *mamA* transcript in the *mamA* KD relative to WT strain were not determined.
- Microscopy of strain *mamA* KD—ΔMSMEG_3214 was not performed.
- Enzymatic activities of MamA and MSMEG_3214 were not determined, and DNA methylation status was not measured.

## Data Availability

All the data supporting the findings is contained within the manuscript.

## Abbreviations

R-M system: Restriction-modification system
ATc: Anhydrotetracycline
KD: Knockdown
DIC microscopy: Differential interference contrast microscopy
CRISPRi: Clustered regularly interspaced short palindromic repeats interference
PAM: Protospacer adjacent motif
CFUs: Colony-forming units
OD: Optical density
